# Older German adults’ health-related quality of life and associated social factors: Lina Spagert

**DOI:** 10.1093/eurpub/ckac130.033

**Published:** 2022-10-25

**Authors:** C Geigl, L Spagert, J Loss, M Leitzmann, C Janssen

**Affiliations:** 1 Department of Applied Social Sciences, Munich University of Applied Sciences, Munich, Germany; 2 Department of Epidemiology and Preventive Medicine, University of Regensburg, Regensburg, Germany; 3 Department of Engineering and Management, Munich University of Applied Sciences, Munich, Germany; 4 Department of Epidemiology and Health Monitoring, Robert Koch Institute, Berlin, Germany

## Abstract

**Background:**

A differentiated analysis of the structural relationships between social factors and health-related quality of life (HRQOL) in older German adults has not yet been conducted. In this analysis, we aimed to examine the relationships between sociodemographic, socioeconomic, psychosocial, and behavioural factors and both physical and mental HRQOL in older German adults.

**Methods:**

A community-based postal survey was used to collect cross-sectional data from German adults aged 65 and older (n = 1687, 33% response proportion, 52% female). Physical and mental dimensions of HRQOL were assessed using Short Form 36, version 2. Multiple linear regression models were used to analyse the associations between social factors and both physical and mental HRQOL.

**Results:**

Health locus of control, physical activity, and income were positively associated with both physical HRQOL (Adj. R2 = 0.34, p < 0.001) and mental HRQOL (Adj. R2 = 0.22, p < 0.001), whereas age was negatively associated with both. Alcohol use was positively associated with physical HRQOL, and social support was positively associated with mental HRQOL.

**Conclusions:**

A differentiated understanding of the relationships between social factors and HRQOL assists in group-specific targeting of health interventions. Demand-oriented interventions should consider underlying social factors to reduce socially determined inequities in HRQOL among older German adults. Depending on the focus of the intervention, it may be helpful to take specific social conditions into account. The results may be transferable to municipalities in high-income European countries.

**Key messages:**

- Sociodemographic, socioeconomic, psychosocial, and behavioural factors are associated with physical and mental health-related quality of life in older German adults.
- Social conditions should be considered when targeting group-specific interventions to reduce socially determined inequities in health-related quality of life among older adults.

